# Developmental transcriptome profiling of bovine muscle tissue reveals an abundant GosB that regulates myoblast proliferation and apoptosis

**DOI:** 10.18632/oncotarget.16644

**Published:** 2017-03-29

**Authors:** Hui Li, Xuefeng Wei, Jiameng Yang, Dong Dong, Yongzhen Huang, Xianyong Lan, Martin Plath, Chuzhao Lei, Xinglei Qi, Yueyu Bai, Hong Chen

**Affiliations:** ^1^ Shaanxi Key Laboratory of Molecular Biology for Agriculture, College of Animal Science and Technology, Northwest A&F University, Shaanxi, Yangling 712100, China; ^2^ Bureau of Animal Husbandry of Biyang County, Biyang, Henan 463700, China; ^3^ Animal Health Supervision of Henan Province, Bureau of Animal Husbandry of Henan province, Zhengzhou, Henan 450008, China

**Keywords:** bovine, muscle, RNA-seq, GosB, apoptosis

## Abstract

The formation of bovine skeletal muscle involves complex developmental and physiological processes that play a vital role in determining the quality of beef; however, the regulatory mechanisms underlying differences in meat quality are largely unknown. We conducted transcriptome analysis of bovine muscle tissues to compare gene expression profiles between embryonic and adult stages. Total RNAs from skeletal muscle of Qinchuan cattle at fetal and adult stages were used to construct libraries for Illumina next-generation sequencing using the Ribo-Zero RNA sequencing (RNA-Seq) method. We found a total of 19,695 genes to be expressed in fetal and adult stages, whereby 3,299 were expressed only in fetal, and 433 only in adult tissues. We characterized the role of a candidate gene (GosB), which was highly (but differentially) expressed in embryonic and adult skeletal muscle tissue. GosB increased the number of myoblasts in the S-phase of the cell cycle, and decreased the proportion of cells in the G0/G1 phase. GosB promoted the proliferation of myoblasts and protected them from apoptosis via regulating Bcl-2 expression and controlling the intracellular calcium concentration. Modulation of GosB expression in muscle tissue may emerge as a potential target in breeding strategies attempting to alter myoblast numbers in cattle.

## INTRODUCTION

The development and growth of skeletal muscle-the primary target of agricultural meat production-is a complex process, and the regulatory mechanisms underlying differences in meat quality are still poorly understood [1, 2]. During prenatal and very early postnatal development, muscle growth in vertebrates depends on an increasing number of muscle fibers (hyperplasia) [3, 4]. Once that growth phase has been completed, numbers of fibers remain constant, but the fiber volume continues to increase (hypertrophy) [3, 4]. As a matter of course, these processes influence the meat quality of livestock [1, 5]. Domestication of wild bovids and subsequent selection for desirable phenotypic traits, crossing, and inbreeding, have led to dramatic phenotypic differences between breeds, with some cattle breeds being optimized for meat or milk production, while others were selected for labor purposes. Our current study was motivated by current problems of the Chinese beef cattle industry that faces problems as numerous traditional Chinese cattle breeds were originally used for labor purposes rather than mean production [2]. Here, we take a first step to explore developmental genetic mechanisms underlying the remarkably high meat quality of Qinchuan cattle. This breed ranks among the top five Chinese yellow cattle breeds in terms of meat quality with a high muscle fat content [2, 6]. It also shows a high tolerance to roughage feeding and general stress resistance [2, 6]. High-throughput RNA sequencing (RNA-Seq) provides a powerful tool to identify genomic variation that is associated with divergent phenotypic traits and thus, allows insights into the molecular mechanisms underlying the domestication process [2, 7]. In this study, we used the Ribo-Zero RNA-Seq method—a novel technology that can capture both coding RNA and noncoding RNA from intact or fragmented RNA samples [8, 9]—to compare whole transcriptomes of bovine embryonic and adult skeletal muscle tissue in unprecedented depth. Our study may serve as a starting point for future research programs comparing different breeds or breeding stocks.

In the second part of our study, we focused on GosB, which was found to be highly (but differentially) expressed in embryonic and adult skeletal muscle tissue (see Results), as a candidate gene and explored its role in cattle muscle development. The activator protein 1 (AP-1) family of transcription factors comprises various combinations of Fos (Fos, GosB, Fra-1, and Fra-2) and Jun proteins (c-Jun, JunB, and JunD) which upon dimer formation regulate myoblast differentiation and influence adipocyte commitment [10, 11]. The AP-1 transcription complex was shown to be involved in muscle differentiation: myocyte enhancer factor 2 (MEF2) is expressed during muscle differentiation and activates the transcription of the c-Jun promoter, which dimerizes with Fra-2. The resulting heterodimer activates the transcription of structural ‘muscle genes’ and myogenic regulatory factors, such as MyoD [12]. Although GosB, as another protein contained in members of the AP-1 family, was found to be highly expressed in bovine skeletal muscle, whether and how GosB plays a role during bovine muscle development is currently not well understood. In *GosB* transgenic mice, GosB stimulates fibroblast proliferation and cell cycle progression by directly or indirectly inducing cyclin D1 transcription [13]. The *GosB* products, especially ΔGosB, appear to regulate cell proliferation, cell differentiation and cell death in rat embryo cell [14–16]. For instance, ΔGosB overexpression activates the proliferation of quiescent Rat-a cells to exit the G1-phase and initiate DNA replication, and induces delayed apoptosis in Rat-a cells dependent on regulating the release of mitochondrial cytochrome c followed by the activation of Caspase-9 and -3 [16]. These observations suggest that GosB has anti-apoptotic and proliferation-enhancing effects, thus regulating cell survival. However, the roles of GosB in regulating bovine myoblast proliferation and apoptosis are poorly understood.

In this study, an extensive and reliable transcriptomic dataset was obtained from embryonic and adult skeletal muscle samples of Qinchuan cattle using the Ribo-Zero RNA-Seq approach. The high-quality sequence data allowed us to identify candidate genes related to muscle development in cattle. We also performed Gene Ontology (GO) analysis and compared differentially regulated genes to genes annotated in the Kyoto Encyclopedia of Genes and Genomes (KEGG) to explore interactions and reaction networks of those genes. We then focused on GosB as a candidate gene to explore its role in myoblast proliferation and apoptosis. We found that GosB promoted myoblasts proliferation and protected the cells from apoptosis by regulating the intracellular calcium concentration. Our study will provide new insights into the genetic mechanisms underlying the exceptional meat quality of Qinchuan cattle, and also provides a foundation to further improve the meat quality of Qinchuan cattle and other Chinese cattle breeds.

## RESULTS

### Gene expression profiles of bovine muscle tissue at different developmental stages

**Figure 1:**
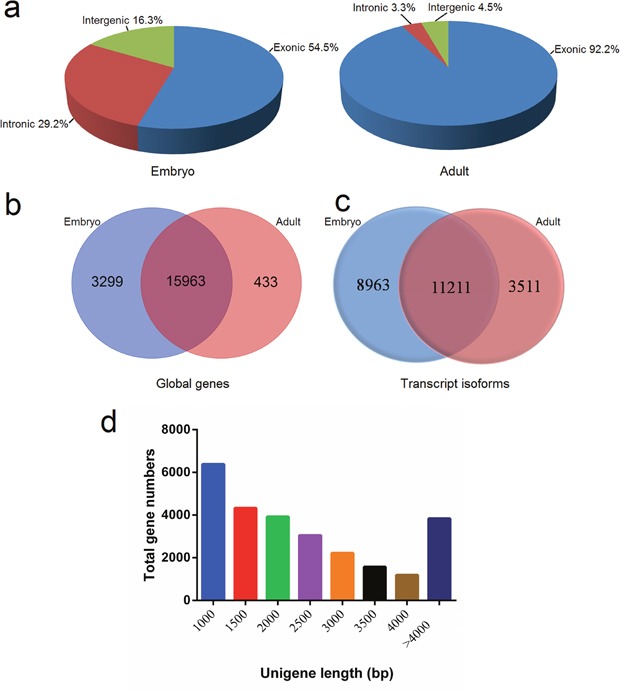
Features of unigenes in bovine muscle tissue **(a)** Genomic origin of bovine muscle unigenes at embryonic and adult stages; Venn diagram of globally transcribed genes **(b)** and transcript isoforms **(c)** expressed at two developmental stages; **(d)** Size distribution of the unigenes.

In total, we obtained 73,229,494 to 89,035,574 and 64,312,418 to 105,185,382 clean reads from the libraries from embryonic tissues (n = 3) and tissues from adult cattle (n = 3), respectively. Filtering and removal of sequence reads with adapters and low quality reads resulted in 50-62 and 54-89 million mapped clean reads, respectively (Table 1). The proportion of mapped sequence reads aligned to exonic regions was markedly lower in embryonic samples (54.5%) than in adult samples (92.2%). Conversely, the percentage of mapped reads aligned to intron regions was dramatically higher at the embryonic stage (29.2%) compared to the adult stage (3.3%; Figure 1a).

**Table 1:** Summary of reads mapping to the bovine reference genome

Samples	Embryo1	Embryo2	Embryo3	Adult1	Adult2	Adult3
**Raw reads**	111,340,382	106,866,166	134,501,982	139,160,360	101,780,186	101,084,070
**Clean reads**	80,480,570	73,229,494	89,035,574	105,185,382	64,312,418	68,886,634
**Mapped reads**	54,979,060	49,922,182	62,333,225	88,546,667	53,810,383	58,188,271
**Mapping ratio**	68.31%	68.17%	70.01%	84.18%	83.67%	84.47%
**Uniquely mapped reads**	50,334,911	45,233,401	57,170,376	86,082,724	52,330,653	56,687,937
**Uniquely mapping ratio**	62.54%	61.77%	64.21%	81.84%	81.37%	82.29%

We found a total of 19,695 genes to be expressed in both the embryo and adult stages, while 3,299 and 433 genes were present only in embryonic and adult stages, respectively (Figure 1b). While the term “gene” conventionally refers to cases of one or a few transcript isoforms that share mature spliced exons, the term “transcript” means any genomic locus transcribed into RNA molecules. Isoforms from the same gene encoding different proteins can have specific functions in the spatio-temporal regulation of developmental processes [8]. In this study, 23,685 transcript isoforms were detected, and 20,174, and 14,722 transcripts were present in the embryonic and adult samples, respectively. Moreover, 8,963, and 3,511 transcripts were specific to the embryonic and adult stages, respectively (Figure 1c). The length of unigenes in this study was assessed through by the following standard metrics: minimum length, maximum length, mean length, median length, *N*_50_-value, and total length (Table 2). The average length of unigenes was 2,373 bp, while the median length was 1,808 bp. The size distribution is shown in Figure 1d; we found that the size of most genes was no more than 3,000 bp.

**Table 2:** Assembly results of unigenes

Item	Unigene	Min length	Mean length	Median length	N50	Max length	Total length
**Numeber**	19,695	201	2,373	1,808	3,166	103,350	62,870,079

Alternative splicing (AS) generates complex proteomes and thus, plays a vital role in the mammalian development and growth [8]. We analyzed alternative splicing dynamics using ASprofile and detected 57,184 to 58,393 and 55,632 to 57,859 splicing events in the libraries from embryonic and adult developmental stages, respectively (Table 3). To further elucidate the mechanisms of alternative splicing, all splicing events were divided into 12 different types, including exon skipping (SKIP) and cassette exons (MSKIP), retention of single (IR) and multiple (MIR) introns, alternative exon ends (AE), alternative transcription start sites (TSS), alternative transcription termination site (TTS), and so forth. Notably, TSS and TTS were the dominant mechanisms of alternative splicing (> 70% of cases) in both embryonic and adult samples.

**Table 3:** Types and numbers of different alternative splicing events

AS category	Embryo 1	Embryo 2	Embryo 3	Adult 1	Adult 2	Adult 3
**AE**	2,443(4.18%)	1,996(3.49%)	2,267(3.89%)	2,772(4.78%)	2,597(4.65%)	2,551(4.59%)
**IR**	1,798(3.09%)	1,968(3.44%)	1,720(2.95%)	918(1.59%)	774(1.39%)	678(1.22%)
**MIR**	258(0.44%)	322(0.56%)	301(0.52%)	81(0.14%)	64(0.11%)	76(0.14%)
**MSKIP**	1,037(1.78%)	872(1.52%)	949(1.63%)	1,256(2.17%)	1,185(2.12%)	1,200(2.16%)
**SKIP**	5,040(8.63%)	4,200(7.34%)	5,068(8.69%)	6,680(11.55%)	6,454(11.56%)	6,506(11.69%)
**TSS**	23,592(40.4%)	23,413(40.94%)	23,760(40.73%)	22,616(39.09%)	21,879(39.18%)	21,799(39.18%)
**TTS**	21,266(36.42%)	21,557(37.70%)	21,349(36.60%)	20,609(35.62%)	20,179(36.13%)	20,130(36.18%)
**XAE**	510(0.87%)	504(0.88%)	516(0.88%)	511(0.88%)	491(0.88%)	462(0.83%)
**XIR**	452(0.77%)	515(0.91%)	445(0.76%)	328(0.57%)	288(0.52%)	277(0.50%)
**XMIR**	110(0.19%)	106(0.19%)	118(0.20%)	74(0.13%)	59(0.11%)	64(0.12%)
**XMSKIP**	703(1.2%)	655(1.15%)	682(1.17%)	711(1.23%)	658(1.18%)	669(1.20%)
**XSKIP**	1,184(2.03%)	1,076(1.88%)	1,159(1.98%)	1,303(2.25%)	1,219(2.17%)	1,220(2.19%)
**Total**	58,393(100%)	57,184(100%)	58,334(100%)	57,859(100%)	55,847(100%)	55,632(100%)

### Identification of differentially expressed genes

**Figure 2:**
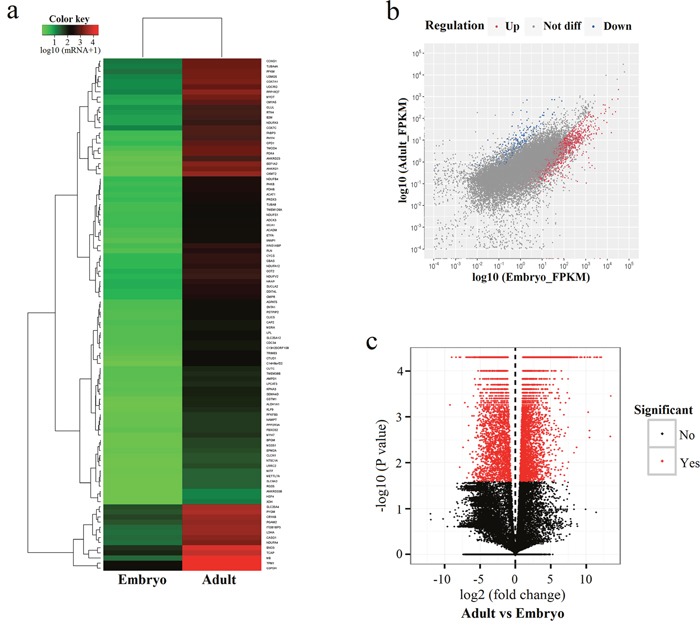
Differentially expressed genes in bovine muscle tissues **(a)** Heat map of 100 most differentially expressed genes comparing embryonic and adult stages; **(b)** Scatter plot showing the correlation of gene expression levels between embryonic and adult stages; **(c)** Volcano plots were constructed using fold-change values and *p*-values. Vertical lines correspond to 2-fold up- and down-regulation when comparing the two developmental stages.

A clustered heatmap of differentially expressed genes along with a Pearson correlation is shown in Figure 2a. To further explore the potential functions of differentially expressed genes, we compared transcript abundances of both developmental stages using a scatter plot (Figure 2b). Pairwise comparison of gene abundances during muscle development exhibited that several genes showed variation in expression patterns, and many genes were more expressed at the embryonic stage.

We found 5,288 unigenes to be significantly differently expressed (*P* < 0.05) at the embryonic and adult stages; all differentially expressed genes are provided in Supplementary Table 2. 4,766 unigenes were up-regulated in samples from adult tissues compared to embryonic samples, while 482 unigenes were down-regulated with at least 2 fold-change in expression levels (Figure 2c). The top 10 unigenes that were up-regulated at the adult stage compared to the embryonic stage are exhibited in Table 4 (FPKM > 2). XLOC_276182 (CKMT2) was the most up-regulated unigene (5,299.06-fold increase in expression at the adult stage compared to the embryonic stage), followed by XLOC_194704 (ANKRD1, 2,846.84-fold increase) and XLOC_054863 (EEF1A2, 2,813.28-fold increase). The top 10 down-regulated differentially expressed genes are shown in Table 5 (FPKM > 2). The most strongly down-regulated unigene was XLOC_119825 (MYL4, 223.38-fold decreas at the adult stage compared to the embryonic stage), followed by XLOC_309473 (ARHGAP36, 106.72-fold decrease). The up-regulated differentially expressed gene with the highest overall level of expression (FPKM = 62083.6) was XLOC_170404 (7SK) with a 4.54-fold increase in expression levels in adult samples, and the down-regulated differentially expressed gene with the highest overall expression level (FPKM = 733.7) was IGF2 with a 26.33-fold decrease in expression levels (Table 6).

**Table 4:** The top 10 up-regulated unigenes at the adult stage compared to the embryonic stage

ID	Annotatedgene	Adult (FPKM)	Embryo (FPKM)	log2(Adult/Embryo)	P-value
**XLOC_276182**	CKMT2	2651.44	0.5	12.37	5.00E-05
**XLOC_194704**	ANKRD1	1571.17	0.55	11.48	5.00E-05
**XLOC_054863**	EEF1A2	1951.63	0.69	11.46	5.00E-05
**XLOC_239696**	PDK4	1414.15	0.93	10.58	5.00E-05
**XLOC_035593**	ANKRD23	689.5	0.564	10.26	5.00E-05
**XLOC_219156**	TMOD4	1370.27	1.54	9.8	5.00E-05
**XLOC_065922**	C14H8orf22	157.43	0.18	9.75	5.00E-05
**XLOC_255920**	MB	15279.7	22.31	9.42	5.00E-05
**XLOC_253590**	GPD1	1065.89	2.87	8.54	5.00E-05
**XLOC_293784**	ALDH1A1	86.81	0.24	8.53	5.00E-05

**Table 5:** The top 10 down-regulated unigenes at the adult stage compared to the embryonic stage

ID	Annotatedgene	Adult (FPKM)	Embryo (FPKM)	log2(Adult/Embryo)	P-value
**XLOC_119825**	MYL4	1.05	233.53	-7.8	5.00E-05
**XLOC_309473**	ARHGAP36	0.41	43.52	-6.74	5.00E-05
**XLOC_155395**	DLK	1.93	195.27	-6.66	5.00E-05
**XLOC_088458**	TNNT2	1.16	58.21	-5.66	5.00E-05
**XLOC_213774**	IGF2	27.87	733.7	-4.72	5.00E-05
**XLOC_255105**	ERBB3	0.4	12.26	-4.93	5.00E-05
**XLOC_251201**	CSDC2	0.35	10.59	-4.93	5.00E-05
**XLOC_177794**	EMILIN2	1.36	28.99	-4.41	5.00E-05
**XLOC_096537**	SFRP2	1.06	21.77	-4.37	5.00E-05
**XLOC_024754**	ACTC1	36.67	724.14	-4.3	5.00E-05

**Table 6:** Differentially and highly expressed genes when comparing embryonic and adult libraries

ID	Annotatedgene	Adult (FPKM)	Embryo (FPKM)	log2(Adult/Embryo)	P-value
**XLOC_170404**	7SK	62083.6	13662.7	2.18396	5.00E-05
**XLOC_063137**	TNNC2	32411.3	2099.25	3.94855	5.00E-05
**XLOC_185879**	MYLPF	30372.1	783.96	5.27582	5.00E-05
**XLOC_025282**	Metazoa_SRP	28180.9	6347.16	2.15053	5.00E-05
**XLOC_025489**	TPM1	23600.8	199.54	6.89	5.00E-05
**XLOC_257641**	G3PDH	17280.7	156.07	6.79	5.00E-05
**XLOC_217773**	TNNI2	16670	746.27	4.48	5.00E-05
**XLOC_255920**	MB	15279.7	22.31	9.42	5.00E-05
**XLOC_213774**	IGF2	27.87	733.70	-4.72	5.00E-05
**XLOC_024754**	ACTC1	36.67	724.14	-4.30	5.00E-05
**XLOC_123373**	MYHC-EMBRYONIC	1.97	703.29	-8.48	2.95E-03
**XLOC_233524**	COL1A2	78.7	630.20	-3	5.00E-05
**XLOC_174413**	HIST1H3A	18.07	273.35	-3.92	5.00E-05
**XLOC_119825**	MYL4	1.05	233.53	-7.8	5.00E-05
**XLOC_155395**	DLK	1.93	195.27	-6.66	5.00E-05
**XLOC_213707**	CDKN1C	35.09	186.9	-2.41	5.00E-05

### Delineation of GO and KEGG pathway analysis

**Figure 3:**
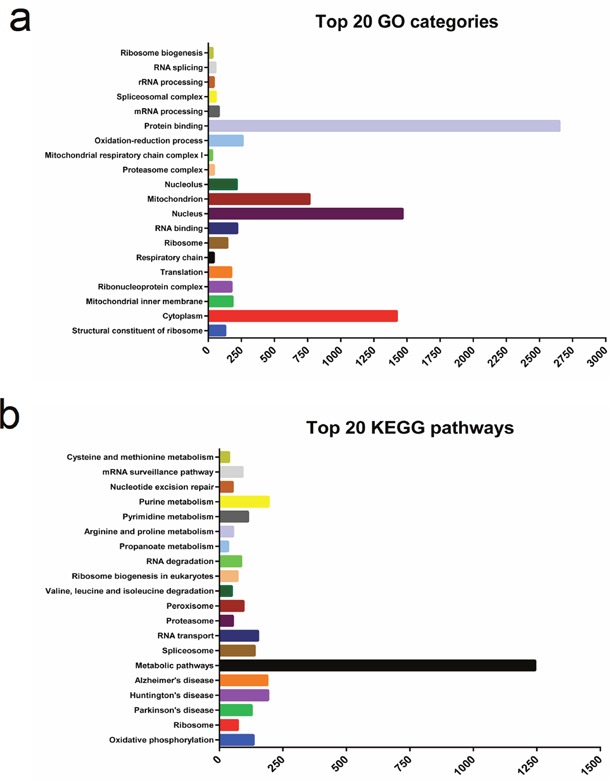
The top 20 Gene Ontology (GO) and Kyoto Encyclopedia of Genes and Genomes pathways (KEGG) of the differentially expressed genes detected in our present study

We performed GO enrichment analysis to further investigate the potential functions of differentially expressed genes in regulating muscle development. In this study, GO enrichment of significantly differentially expressed genes was categorized into 597 functional groups (*P* < 0.05). 157 terms were significantly enriched in ‘molecular function’ (Supplementary Table 3), and the most significantly enriched GO term was ‘structural constituent of ribosome’ (GO: 0003735) with 128 annotated genes, followed by ‘RNA binding’ (GO: 0003723) and ‘protein binding’ (GO: 0005515). In the GO category ‘cellular component’, 153 terms were significantly enriched (Supplementary Table 4). The most significantly enriched GO term was ‘cytoplasm’ (GO: 0005737) with 1,424 annotated genes, followed by ‘mitochondrial inner membrane’ (GO: 0005743), ‘ribonucleoprotein complex’ (GO: 0030529), and ‘translation’ (GO:0006412). 287 GO terms were significantly enriched in ‘biological processes’ and were related to various processes (Supplementary Table 5) such as ‘oxidation-reduction process’ (GO: 0055114), ‘mRNA processing’ (GO: 0006397), and ‘rRNA processing’ (GO: 0006364). The top 20 functional GO annotations for the differentially expressed genes detected in our study are shown in Figure 3a.

We used KEGG pathway enrichment analysis to shed additional light on the biological functions and molecular interactions of differentially expressed genes. We found 224 pathways to be significantly enriched, and the highest level of significance was found for ‘oxidative phosphorylation’ (ko00190) with 95 annotated genes, followed by ‘Ribosome’ (ko03010, 60 genes) and ‘Parkinson's disease’ (ko05012, 85 genes). The top 20 significantly enriched KEGG pathways are shown in Figure 3b. Our results suggest that these pathways may be involved in the development and growth of skeletal muscle.

### Identification of GosB as a candidate gene

**Figure 4:**
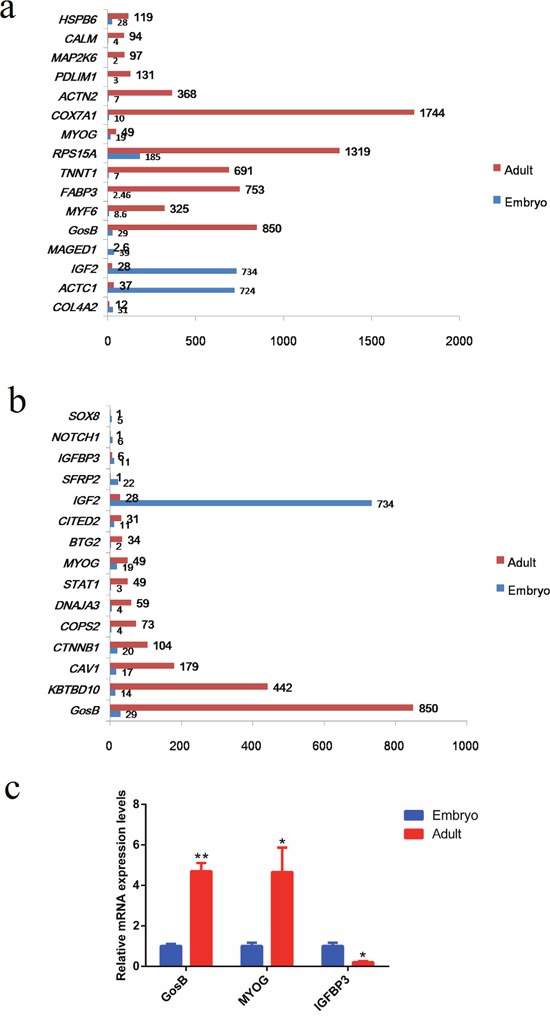
Identification of GosB as a candidate gene to explore its role in mammalian muscle development **(a)** The expression levels of 16 genes exhibited significantly different expression levels during bovine muscle development; **(b)** The expression levels of 16 genes exhibited significantly different expression about cell proliferation and differentiation; **(c)** Validation of different expression levels (mRNA concentrations) of *GosB*, *MyoG* and *IGFBP3* by means of real-time PCR. *P < 0.05; **P < 0.01.

Based on the analyses of both GO terms and KEGG pathways, several differentially expressed genes were significantly enriched (*P* < 0.0001) in the process of muscle development (GO: 0006936, GO: 0014733, GO: 0014809, GO: 0048738, ko04260, ko04270). According to these results, we randomly selected 16 differentially expressed genes that exhibited highly significantly different expression levels in the two developmental stages of bovine muscle tissue (*COL4A2*, *ACTC1*, *IGF2*, *MAGED1*, *GosB*, *MYF6*, *FABP3*, *TNNT1*, *RPS15A*, *MYOG*, *COX7A1*, *ACTN2*, *PDLIM1*, *MAP2K6*, *CALM*, *HSPB6*; Figure 4a). Moreover, the proliferation and differentiation of myoblasts are fundamental processes of skeletal muscle formation. Hence, we also analyzed genes related to muscle cell proliferation and differentiation, and found 46 genes to exhibit significantly different expression levels when comparing the two developmental stages (Supplementary Table 6). As can be seen Figure 4b, GosB was one of the most significantly differentially expressed genes. To confirm our RNA-Seq results, we performed quantitative real-time PCR (qPCR) to quantify the mRNA concentrations of *GosB*, *MyoG* and *IGFBP3* at the embryonic and adult developmental stages (Figure 4c). We found the expression of GosB to be much higher (~4.7 fold) at the adult stage compared with the embryonic stage. Therefore, we chose GosB, which is known to be involved in cell proliferation, differentiation and apoptosis [13–15], as a candidate gene to further explore its role in cattle muscle development.

**Figure 5:**
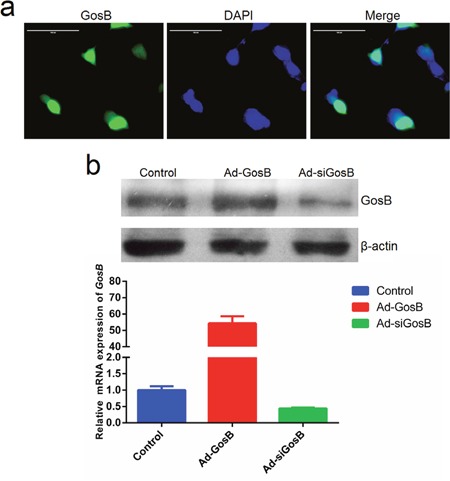
Characterization of GosB in murine C2C12 myoblast **(a)** Sub-cellular localization of GosB in C2C12 cells. Scale bar indicates 100 μm. **(b)** Relative mRNA levels of GosB in C2C12 cells transfected with Ad-GosB or Ad-siGosB for 48h, and representative image of western blot analysis.

In order to understand the role of GosB in myoblast proliferation and differentiation, the GosB overexpression recombinant adenovirus Ad-GosB and interference recombinant adenovirus Ad-siGosB were constructed. The murine C2C12 myoblast cell line is a useful tool to study the proliferation and differentiation of myoblasts and to explore regulatory pathways [17]. When we investigated the localization of GosB in C2C12 cells, we found GosB to target to the cell nucleus, suggesting that it may act as a transcription factor (Figure 5a). GosB overexpression markedly increased the expression of GosB, and GosB expression was significantly reduced in cells transfected with Ad-siGosB (*P* < 0.01; Figure 5b).

### Effects of GosB on myocyte differentiation and proliferation

To establish the involvement of GosB in myoblast differentiation, the expression of GosB was quantified in C2C12 cells and primary bovine myoblasts after differentiation had been induced for 0, 1, 2, 4, and 6 days. We found that *GosB* was down-regulated during myoblasts differentiation (Supplementary Figure 1). We used qPCR on mRNA extracted from cells transfected with Ad-GosB or Ad-siGosB to establish expression patterns of two marker genes of myoblast differentiation, *MyoD* and *MHC*. However, we found that the expression of both genes was not statistically different between treatment groups (*P* > 0.05, Supplementary Figure 1), suggesting that GosB is not directly involved in myoblast differentiation, and may rather regulate muscle cell proliferation to affect muscle development, as a cell survival-enhancing effect was also reported from other cell types [13–16].

**Figure 6:**
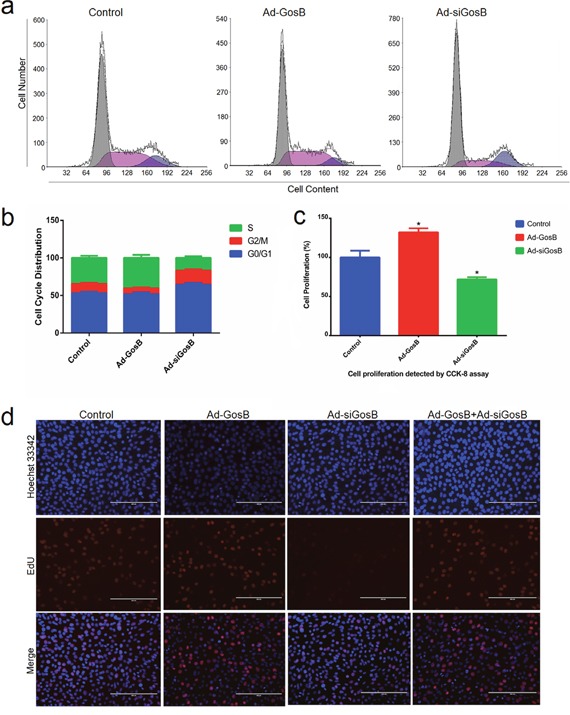
Effects of GosB on aspects of the cell cycle and cell proliferation Murine C2C12 cells were transfected with Ad-GosB or Ad-siGosB for 48 h, and subsequently subjected to cell cycle assay using a flow cytometer and cell proliferation analysis using cell counting kit-8 (CKK-8) and EdU incorporation assays. Scale bar indicates 200 μm. *P < 0.05.

To assess the role played by GosB during cell proliferation, we again pretreated C2C12 cells with Ad-GosB or Ad-siGosB. Cell cycle analysis revealed that GosB overexpression led to a higher number of C2C12 cells entering the S-phase, while decreasing the proportion of cells in the G0/G1 phase (Figure 6a, 6b). When GosB expression was knocked down by treatment with Ad-siGosB, the number of C2C12 cells in the G0/G1 phase was higher. We found Ad-siGosB to inhibit cell proliferation while GosB overexpression promoted cell proliferation according to the Cell Counting Kit-8 (CCK-8) and EdU incorporation assays (Figure 6c, 6d). We also found GosB inhibited cell proliferation in primary bovine myoblasts as demonstrated in C2C12 cells (Supplementary Figure 2).

### Effects of GosB on apoptosis of myocytes

**Figure 7:**
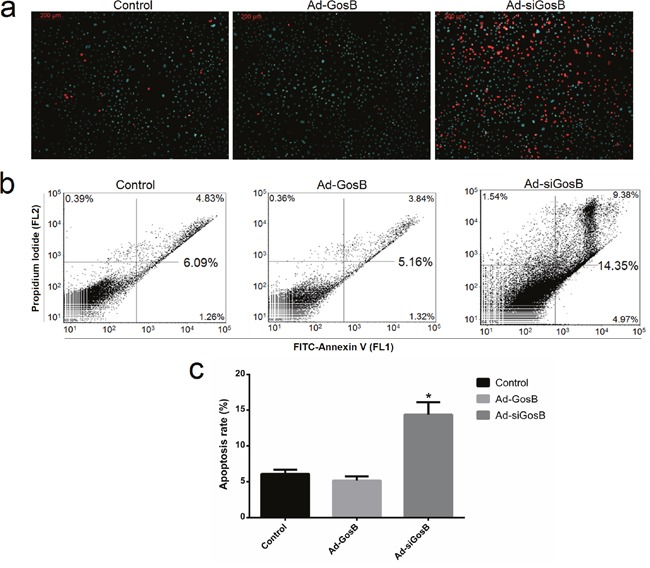
Effects of GosB on cell apoptosis Cell apoptosis was determined by Hoechst 33342/PI dual staining assays **(a)** and Annexin V-FITC/PI binding followed by flow cytometry **(b, c)**. Data are shown as means ± SEM for three individuals. *P < 0.05.

**Figure 8:**
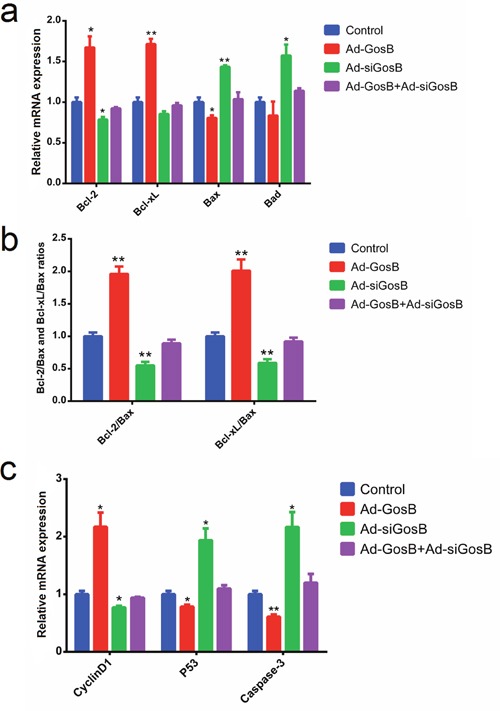
Effects of GosB on the expression of cell survival related genes in murine C2C12 cells C2C12 cells were transfected with Ad-GosB or Ad-siGosB for 48 h, and mRNA levels of *Bcl-2*, *Bcl-xL*, *Bax*, *Bad*, *CyclinD1*, *p53*, *Caspase3* analyzed by means of quantitative real time PCR (qPCR). Bcl-2/Bax and Bcl-xL/Bax ratios are also shown. Data are depicted as means ± SEM for three individuals. *P < 0.05; **P < 0.01.

FITC-AnnexinV/propidium iodide (PI) and Hoechst 33342/PI staining assays showed Ad-siGosB induced myoblasts apoptosis, while GosB overexpression protected cells from apoptosis (Figure 7 and Supplementary Figure 3). Thus, our results confirm that GosB has a survival-enhancing effect in C2C12 cells. Bcl-2 has been described as an anti-apoptotic protein, and plays important roles in regulating proliferation and apoptosis in myoblasts [18, 19]. We asked whether Bcl-2 is involved in the survival-enhancing effects of GosB in myoblast, and thus quantified the expression of Bcl-2 in C2C12 cells and primary bovine myoblasts pretreated with Ad-GosB or Ad-siGosB using qPCR. We found GosB to promote the expression of *Bcl-2*, and to significantly increase *Bcl-xL* expression while suppressing the expression of *Bax* and *Bad* (Figure 8a and Supplementary Figure 3). We also found GosB overexpression to lead to increase the Bcl-2/Bax and Bcl-xL/Bax ratios (Figure 8b). Moreover, we found GosB to result in increased *CyclinD1* expression and to inhibit the expression of *p53* and *Caspase-3* (Figure 8c). Taken together, these observations suggest that the anti-apoptotic effect of GosB in myoblasts is mediated by the expression of Bcl-2.

**Figure 9:**
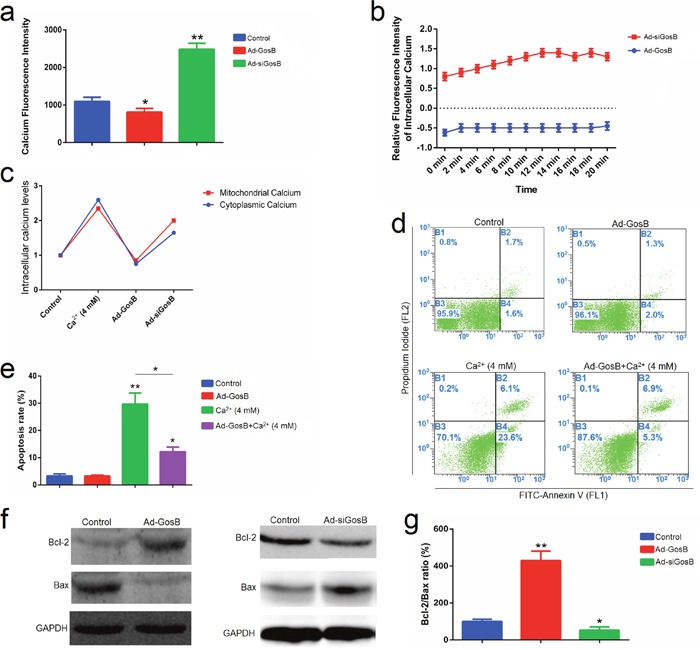
GosB prevents mitochondrial calcium overload and subsequent apoptotic events **(a, b)** Confocal laser scanning microscopic examination of C2C12 cells treated with Rhod-2 AM showing mitochondrial Ca^2+^; **(c)** Cytosolic and mitochondrial calcium modulation was studied using Fluo-3 AM and Rhod-2 AM, respectively, using flow cytometry; **(d, e)**; C2C12 cells were transfected with Ad-GosB for 48 h, and subsequently exposed to 4 mM CaCl_2_ for 24 h. Cell apoptosis was determined by Annexin V-FITC/PI binding followed by flow cytometry; **(f)** Protein levels of Bcl-2 and Bax detected by Western blot analysis; **(g)** Bcl-2/Bax protein ratios. Values are means ± SEM for three individuals. *P < 0.05; **P < 0.01.

Activation of intracellular Ca^2+^ triggers cellular dysfunction and cell death [20, 21], while GosB promotes calcium deposition [22, 23]. Thus we examined cytosolic and mitochondrial calcium levels in C2C12 cells after transfection with Ad-GosB or Ad-siGosB for 48 h. GosB was found to regulate cytosolic and mitochondrial Ca^2+^ concentrations in C2C12 cells (Figure 9a-9c). GosB overexpression decreased cytosolic and mitochondrial calcium concentrations. Ad-siGosB led to a transient increase in calcium concentrations, followed by a relatively long lasting calcium surge. Cell apoptosis induced by acute increases in intracellular Ca^2+^ concentration was abolished by GosB overexpression (Figure 9d, 9e). Calcium overload subsequently activated the apoptotic cascade as evidenced by the inhibition of Bcl-2 and activation of Bax (Figure 9f, 9g). These results demonstrate that GosB protects C2C12 cells from apoptosis by a Ca^2+^-sensitive mechanism.

## DISCUSSION

Comparative transcriptome analyses of tissues at different developmental stages can provide valuable insights into the question of how regulatory gene networks control specific biological processes and how diseases can arise [24, 25]. High-throughput sequencing has rapidly gained popularity for transcriptome analyses in domestic animals due to its sensitivity in detecting not only transcripts in general, but also mRNA isoforms, and to generate quantitative information on the expression levels of annotated genes [2, 4, 8, 26]. RNA-Seq libraries, polyA-Seq, and Ribo-Zero RNA-Seq are the most commonly employed methods to obtain transcriptomic information [25]. In this study, total RNAs from Qinchuan cattle skeletal muscle at fetal and adult stages were used to construct libraries for Illumina next-generation sequencing using the Ribo-Zero RNA sequencing (RNA-Seq) method. At present, Ribo-Zero RNA-Seq is the most sensitive method to study transcriptomes, particularly for cattle with only incompletely annotated genomes being available [8]. Compared with RNA-Seq and polyA-Seq, Ribo-Zero RNA-Seq can capture poly(A)-transcripts from intact and fragmented RNA, and the method appears to be highly technically reproducible [25, 27]. In cattle, muscle fiber numbers increase during prenatal and very early postnatal development, after which fiber numbers stop to increase, while fiber volume still increases [3, 4]. We assessed gene expression patterns at two different developmental stages to gain insights into the molecular mechanisms involved in mammalian skeletal muscle development [8] and later focused on the role of GosB in myocyte proliferation, differentiation, and apoptosis. GosB has been reported to regulate cell proliferation, differentiation and cell death [14–16]. For example, GosB protects rat embryo cells from apoptosis and thus, leads to increased cell survival. It seems that this effect is mediated by the action of GosB in the mitochondrial apoptotic pathway, which is dependent on Caspase-3 and -9 [16]. Consistent with this, GosB in our study promoted the proliferation of murine C2C12 cells and primary bovine myoblasts according to the CCK-8 and EdU incorporation assays. Furthermore, GosB protected myoblasts from apoptosis and increased Bcl-2 expression and decreased the expression of Bax and *Caspase-3*, suggesting that the survival-enhancing role of GosB in myoblasts is dependent on the expression of Bcl-2.

Calcium is a well-known intracellular messenger that modulates many aspects of cell life [28–30]. Intracellular Ca^2+^ accumulation activates the mitochondrial apoptotic pathway with high levels of Bax and activation of Caspase-3 [31–33]. GosB is known to function as a physiologically important regulator of osteoblast differentiation and increases bone formation and bone mass, leading to osteosclerosis [22, 23]. Therefore, we speculated that GosB may adopt its anti-apoptotic effect through regulating intracellular Ca^2+^ concentrations. This notion was supported by the results that GosB overexpression decreased the mitochondrial Ca^2+^ concentration (i.e., fluorescence intensity) at lower levels. Proteins in the Bcl-2 family play a vital role in controlling intracellular Ca^2+^ signals because of their ability to affect—either directly or indirectly—Ca^2+^ storage in the endoplasmic reticulum by interacting with the inositol 1,4,5-trisphosphate receptor [32, 34]. As an anti-apoptotic gene, Bcl-2 inhibits calcium release from the endoplasmic reticulum and mitochondrial calcium uptake, whereas Bax—a member of the pro-apoptotic Bcl-2 family—induces calcium release from the endoplasmic reticulum as well as mitochondrial Ca^2+^ uptake [31, 32, 35]. In our present study, GosB increased the expression of Bcl-2, inhibited cytosolic and mitochondrial calcium accumulation, and protected C2C12 cells from apoptosis induced by acute increases in intracellular Ca^2+^ concentrations. Therefore, our results suggest that GosB can promote myoblasts survival by regulating the intracellular Ca^2+^ concentration-dependent Bcl-2 expression.

In summary, we analyzed the expression profiles of cattle skeletal muscle tissue samples taken at embryonic and adult developmental stages. A large number of differentially expressed genes were detected when comparing both stages. We further characterized and functionally evaluated a differentially expressed gene, GosB. Our findings demonstrate for the first time that GosB promotes myoblast proliferation and protects the C2C12 cells from apoptosis by regulating intracellular calcium concentrations. Thus, modulation of GosB expression in muscle tissue may emerge as potent tool to control myoblast number in the cattle.

## MATERIALS AND METHODS

### Sample preparation

All Qinchuan cattle received humane care as described in the proclamation No. 5 of the Ministry of Agriculture, P.R. China. All experimental procedures used in this study were approved by the Animal Care and Use Committee of the Northwest A&F University. We collected six musculus longissimus tissue samples of Qinchuan cattle from two developmental states each (3 fetal samples, 90 days after fertilization, and 3 adult samples, 24 months after birth) from a local slaughterhouse in Xi’an, P.R. China. Tissue samples were snap-frozen in liquid nitrogen and stored at –80°C for RNA extraction.

### Library preparation and Illumina sequencing

Total RNA of fetal and adult longissimus muscle was isolated using TRIzol reagent (Invitrogen, Carlsbad, CA, USA) following the manufacturer's instructions. We assessed total RNA yield by electrophoresis on a denaturing agarose gel and quantified RNA concentrations using a NanoDrop spectrophotometer (Nano-Drop Technologies, Wilmington, DE, USA) and Agilent 2100 Bioanalyzer. A total of 3 μg RNA per sample was treated with the epicentre Ribo-Zero*™* Kit (Epicentre, Madison, WI, USA) to remove rRNA before constructing the RNA-seq libraries. Afterwards, the RNA samples were fragmented and used to synthesize first- and second-strand complementary DNA (cDNA) with random hexamer primers, dNTPs and DNA Polymerase I according to the manufacturer's manual. The cDNA fragments were cleaned and concentrated using AMPure XP beads (Beckman Coulter, Brea, CA, USA), then the ends were repaired and modified with T4 DNA polymerase and Klenow DNA polymerase to add a single A base and ligate the adapter at the 3′ end of the DNA fragments. The ligated cDNA products were purified with AMPure XP beads and treated with uracil DNA glycosylase (NEB, Ipswich, MA, USA) to remove the second-strand cDNA. Purified first-strand cDNA was enriched with 12 PCR cycles to create the final cDNA library. We checked the quality of the libraries using Agilent 2100 Bioanalyzer (Agilent, Santa Clara, CA) and sequenced them using Illumina HiSeq 2500 Technology (LC Sciences, Houston, TX, USA) with a 150 bp paired-end run.

### Analysis of sequence data

From the raw FASTQ files, adapters were removed using Trim Galore (http://www.bioinformatics.babraham.ac.uk/projects/trim_galore/) and filtered data were aligned to the *Bos taurus* reference genome (bosTau7) from UCSC (http://genome.ucsc.edu/) with TopHat2 (version 2.0.14. Linux_x86_64) [36]. Assembly of linear transcripts and abundance estimations were performed using Cufflinks, which was outsourced to LC-Bio (Hangzhou, China). The assembled transcripts were mapped to the reference annotation using the program Cuffcompare (Cufflinks, v2.2.1) to detect novel genes. Transcript abundances were normalized as the number of uniquely mapped fragments per kilobase of exon per million fragments mapped (FPKM). The raw sequencing dataset supporting the results of this study were deposited at NCBI's Gene Expression Omnibus Database (http://www.ncbi.nlm.nih.gov/geo/). The data are accessible through GEO Series accession number GSE86847 (http://www.ncbi.nlm.nih.gov/geo/query/acc.cgi?acc=GSE86847).

### Gene ontology and pathway analysis

We used GO analysis (http://www.geneontology.org) to annotate differentially expressed genes in our genome-wide expression analyses. GO-terms provide information regarding the biological processes of a gene, either a cellular component or metabolic pathway, and highlight the molecular function(s) while reducing complexity. We used the software TM4 (http://www.tm4.org/mev.html) to screen for differentially expressed genes and to conduct cluster analysis. We also performed KEGG (http://www.kegg.jp) pathway analysis to provide insights into molecular interactions and reaction networks in which differentially regulated genes are involved using DAVID (version 6.7, http://david.abcc.ncifcrf.gov). The -log_10_ *p*-value in this analysis denotes significant enrichment of GO-terms and KEGG pathways in the case of differentially up- and down-regulated entities.

### Adenovirus generation

The cDNA of bovine GosB (GenBank ID no. NM_001102248; https://www.ncbi.nlm.nih.gov/nuccore/NM_001102248.1) was cloned from Chinese Qinchuan cattle longissimus muscle using the following primers: sense 5′- TGTGCCCAGGGAAATGTTT-3′ and antisense 5′-GTCTAAAGCTCACAGAGCAAGAAG-3′. The coding sequences of GosB was subcloned into the pAdTrack-CMV plasmid vector between the *Xho*I and *Scal*I (TaKaRa, Dalian, China) restriction sites to construct a recombinant shuttle vector pAdTrack-CMV-GosB. Then this vector was homologously recombined with plasmid pAdEasy-1 to generate adenoviral plasmid in BJ5183 cells. The adenoviral plasmids linearized by *Pac*I (TaKaRa, Dalian, China) was transfected into 293A cells to generate the adenovirus Ad-GosB. For interference recombinant adenoviruses Ad-siGosB, the BlockiT shRNA interference system was used in this experiment. We designed and synthesized one pair of complementary single-strand DNA oligonucleotides (shRNA-GosB: GATCAGCTAGAGGAAGAAA) which targeting GosB mRNA and then oligonucleotides were cloned into shuttle vector pENTR/CMV-GFP/U6. After detection of interference efficiency, we recombined a pENTR/CMV-GFP/U6-GosB and adenovirus backbone vector pAD/PL-DEST, to produce recombinant vector pAD/PL-DEST/CMV-GFP/U6-GosB. The fifth generation recombinant adenovirus particle (Ad-siGosB) was produced and further amplified by transfecting 293A cells. The titer of adenovirus reached 1.58×10^9^ PFU/mL determined by TCID50 assays. The whole process for adenovirus generation and proliferation was carried out as previously described [37, 38]. The myoblasts at about 80% confluence were transfected with adenovirus supernatant with multiplicity of infection (MOI) = 200.

### Cell culture and treatment

We purchased murine C2C12 myoblasts (ATCC number: CRL-1772™); details on cell culture conditions were described previously [39]. Primary bovine myoblasts were isolated and cultured from bovine longissimus muscle as described in a previous study [40]. Myoblasts at the stage of 80% confluence were plated at 5 × 10^5^ cells/well in six-well plates or 1 × 10^4^ cells/well in 96-well plates (NEST, Wuxi, China) and incubated as described previously [40]. After growth to approximately 80% confluence, the cells were treated with: Ad-GosB (MOI = 200), Ad-siGosB (MOI = 200) or Ca^2+^ (4 mM). After incubation, the myoblasts were used for the different assays outlined below. To induce differentiation of myoblasts, the culture medium was changed to high-glucose Dulbecco's modified Eagle's medium (DMEM) with 2% horse serum [40].

### Total RNA extraction and quantitative real-time PCR

To confirm different expression of GosB in our different treatment groups, we used qPCR. Cells were lysed in 1 mL of Trizol reagent (Invitrogen, Carlsbad, CA). Proteins were removed by adding 200 μL of chloroform. Total RNA was precipitated with an equal volume of isopropanol, and the RNA pellet was washed two times with 75% ethanol. cDNA was synthesized using a transcript first-strand cDNA synthesis kit (TaKaRa, Dalian, China). qPCR primers were shown in Supplementary Table 1. Glyceraldehyde phosphate dehydrogenase (*GAPDH*) was used as housekeeping gene. All qPCR measurements were replicated three times in a Bio-Rad master cycler using the SYBR Green PCR Master Mix (Takara, Dalian, China) according to the manufacturer's protocol. We used the 2^−ΔΔCt^ method to analyze relative expression levels of qPCR data.

### Cell cycle assay

To gain insights into the potential effects of GosB on aspects of the cell cycle, we analyzed the different treatment groups using the Cell Cycle Testing Kit (Multisciences, Hangzhou, China). We harvested cells that had been cultivated in six-well plates and centrifuged them at 800 g/min for 5 min. The supernatant was discarded, and the cells were washed once with cold phosphate buffered saline (PBS). We resuspended the cells in 1 mL of kit reagent A and 10 μL of reagent B, followed by vortexing for 10 s and incubation for 30 min at room temperature, after which the cell suspension was used for flow cytometry (FACS Canto™ II, BD BioSciences, USA).

### Cell proliferation assay

We examined cell proliferation using the CCK-8 assay (Multisciences, Hangzhou, China) and EdU incorporation assay (Ribobio, Guangzhou, China). For the CCK-8 assay, the cells were plated into 96-well culture plates at a density of 1 × 10^4^ cells/well in 100 μL of culture medium per well, and each treatment group had six independent replicates. After 48 h of incubation at 37°C, 10 μL of CCK-8 reagent was added to each well and incubated at 37°C for 2 h. The absorbance of each sample at 450 nm wavelength was detected using a microplate reader (Molecular Devices, Sunnyvale, USA). We also assessed cell proliferation using the Cell-Light EdU DNA cell proliferation kit according to the manufacturer's instructions, with three independent replicates per treatment group.

### Measurement of apoptosis using Annexin V-FITC/PI staining assays

After incubation, cells from the different treatment groups were washed with PBS three times, harvested by trypsinization, washed again with PBS and then resuspended in 500 μL 1 × binding buffer. Then cells were incubated for 10 min in the dark at room temperature in the presence of Annexin V-FITC (5 μL) and PI (10 μL, Vazyme, China). Afterwards, cells were analyzed using flow cytometry (see above), and each treatment group had three independent replicates.

### Measurement of apoptosis using Hoechst 33342/PI dual staining assays

Cell apoptosis was also assessed with Hoechst 33342 and PI double staining (Solarbio, Beijing, China). In brief, after transfection with Ad-GosB or Ad-siGosB for 48 h, cells were washed with PBS and subsequently stained with Hoechst 33342 (100 μg/mL) for 15 min at room temperature. Afterwards, cells were washed with PBS twice, and PI (100 μg/mL) was added before cells were incubated for 10 min at room temperature. Presence or absence of a fluorescence signal was observed under a fluorescence microscope (DM5000B, Leica, Germany). Hoechst 33342^−^/PI^−^ cells (viable cells) showed light blue coloration, Hoechst 33342^+^/PI^−^ cells (early apoptotic cells) showed blue fragmentations, Hoechst 33342^−^/PI^+^ cells (late apoptotic cells) had red fragmentations, and PI^+^ cells (necrotic cells) showed signs of decay.

### Western blot analysis

We collected cells from the different treatment groups, pelleted them by centrifugation and lysed them in RIPA buffer. Total protein was prepared and protein concentration determined using the Bradford method. Proteins were then separated by SDS-polyacrylamide gel electrophoresis and subsequently transferred to nitrocellulose membranes, blocked with milk powder solution for 1.5 h at room temperature, followed by overnight incubation with the primary antibody. Anti-GAPDH was purchased from Bioss biotechnology (Bioss, Shanghai, China). Anti-GosB, anti-Bcl-2, anti-β-actin and anti-Bax were purchased from (Abcam, Cambridge, MA). Then the membranes were washed with PBS-tween and incubated for 1.5 h with horseradish peroxidase-conjugated secondary antibodies (Abcam, Cambridge, MA). Protein bands were detected after treatment with SuperSignal West Femto agent of Thermo (Thermo Scientific, Karlsruhe, Germany).

### Measurement of intracellular calcium

Activation of intracellular Ca^2+^ triggers cellular dysfunction and cell death [20, 21], and GosB promotes calcium deposition [22, 23]. Thus we examined the cytosolic and mitochondrial calcium levels in myoblasts transfected with Ad-GosB or Ad-siGosB. Intracellular calcium trafficking of the cytoplasm and mitochondria were detect using Fluo-3 AM and Rhod-2 AM (2μM, Molecular probes, Life Technologies), respectively. Cells were treated as described previously [41, 42], and screened by means of flow cytometry and use of a confocal laser scanning microscope (Leica, Solms, Germany).

### Statistical analyses

Generally, results in our study were expressed as mean ± standard error (SEM) whenever at least three independent runs were conducted per treatment group. The statistical significance of different RNA expression levels was analyzed by means of a one-way analysis of variance (ANOVA), followed by Tukey's post hoc tests using SPSS 19.0 (Chicago, USA). *P* < 0.05 was considered statistically significant and indicated with one asterisk, while two asterisks indicated *P* < 0.01.

## SUPPLEMENTARY MATERIALS FIGURES AND TABLES













## Abbreviations

AE: alternative exon ends
AP-1: activator protein 1
AS: alternative splicing
CCK-8: cell counting kit-8
cDNA: complementary DNA
DMEM: Dulbecco's modified Eagle's medium
FPKM: fragments per kilobase of exon per million fragments mapped
GAPDH: glyceraldehyde phosphate dehydrogenase
GO: Gene Ontology
IR: retention of a single intron
KEGG: Kyoto Encyclopedia of Genes and Genomes
MEF2: myocyte enhancer factor 2
MIR: retention of multiple introns
MSKIP: cassette exons
PBS: phosphate buffered saline
PI: propidium iodide
qPCR: quantitative real-time PCR
RNA-Seq: RNA sequencing
SEM: standard error of the means
SKIP: exon skipping
TSS: alternative transcription start site
TTS: alternative transcription termination site
XAE: approximate AE
XIR: approximate IR
XMIR: approximate MIR
XMKIP: approximate MSKIP
XSKIP: approximate SKIP
