# Supplementary material for: Developmental transcriptome profiling of bovine muscle tissue reveals an abundant GosB that regulates myoblast proliferation and apoptosis
Source: Oncotarget. 2017 Mar 29;8(19):32083–100. doi: 10.18632/oncotarget.16644 (PMC5458270; doi:10.18632/oncotarget.16644)
Supplement: Supplementary file 1 [file oncotarget-08-32083-s001.pdf]

Developmental transcriptome profiling of bovine muscle tissue reveals an abundant GosB that regulates myoblast proliferation and apoptosis

SUPPLEMENTARY FIGURES AND TABLES

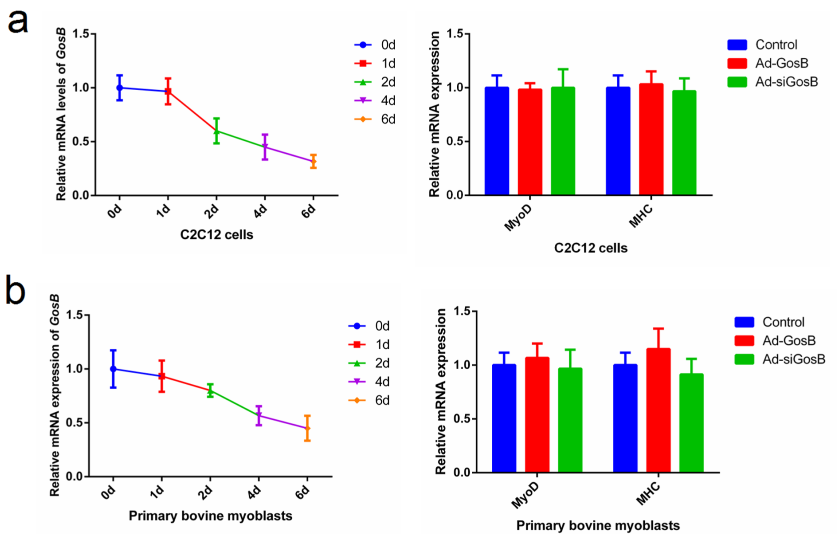

Supplementary Figure 1: GosB in myoblasts differentiation.

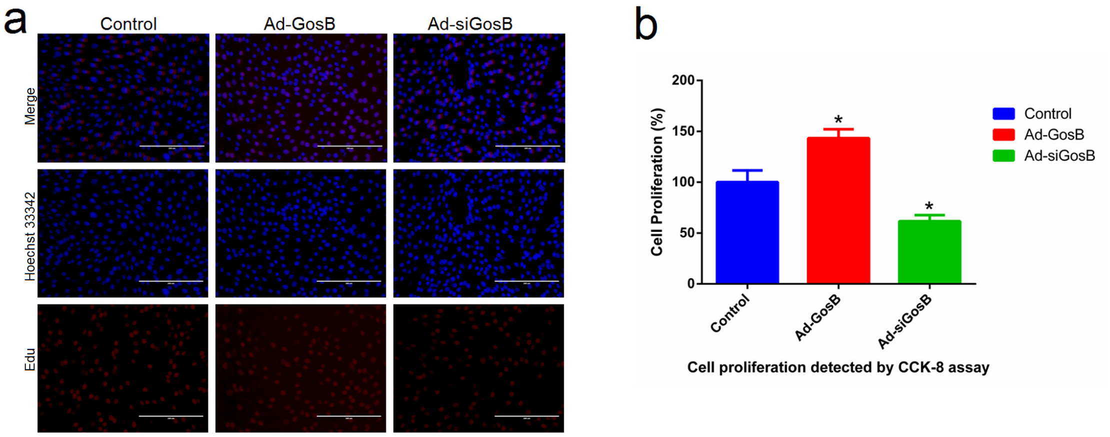

Supplementary Figure 2: Effects of GosB on primary bovine myoblasts proliferation.

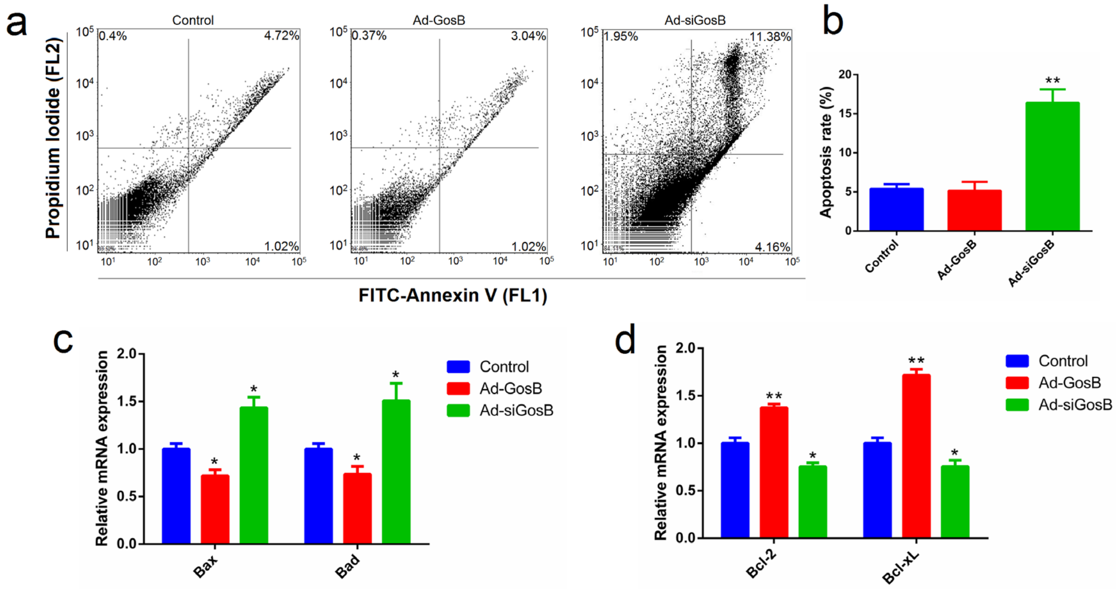

Supplementary Figure 3: Effects of GosB on cell apoptosis in primary bovine myoblasts.

Supplementary Table 1: Primers designed for quantitative real time PCR (qPCR)

Gene	Primer sequence[1] (5′-3′)	Product length (bp)	Efficiency[2]
GosB	F: ACCCACCCTCATCTCTTCCAT<br>R: GCCACTGCTGTAGCCACTCA	130	2.14
GAPDH[3]	F: GCAAGTTCCACGGCACAG<br>R: GGTTCACACCAATCACAA	249	2.16
GAPDH[4]	F: AGGACACTTGTCATCTCATTC<br>R: TTGGAGTCAGGTTCTCTCTTGTA	169	2.18
MYOG	F: CAAATCCACTCCCTGAAA<br>R: GCATAGGAAGAGATGAACA	93	2.13
MYOD[3]	F: GGAAGGGAAGAGCAGAAG<br>R: AAGGACTACAACAACAACAAC	82	1.98
MYOD[4]	F: CCGCTCCGCGACGTAGACTTGA<br>R: GAGTCGAAACACGGGTCATCATA	92	2.03
MHC[3]	F: CAATAAACTGCGGGCAAAGAC<br>R: CTTGCTCACTCCTCGCTTTCA	75	2.01
MHC[4]	F: TGCTCATCTCACCAAGTTCC<br>R: CACTCTTCACTCTCATGGACC	150	1.99
IGFBP3	F: GCGCCCTTACCTGCTACCGT<br>R: GCTGTCCTTGGCATGACCTTTCT	175	2.16
P53	F: TGAACCGCCGACCTATCC<br>R: GCAGGCACAAACACGAACC	95	2.1
Bcl-xL	F: TCGGGATGGAGTAAACTGGG<br>R: CCACGCACAGTGCCCC	65	2.2
Bad	F: GCAGCCACCAACAGTCATCAT<br>R: CAAACTCATCGCTCATCCTTCG	202	1.87
cyclinD1	F: TCTACACTGACAACTCTATCCG<br>R: TAGCAGGAGAGGAAGTTGTTGG	304	1.76
Caspase-3	F: TGTCATCTCGCTCTGGTACG<br>R: AAATGACCCCTTCATCACCA	201	2.02
Bcl-2	F: GGTGGTGGAGGAACTCTTCA<br>R: CAGATGCCGGTTCAGGTACT	160	2.23
Bax	F: GAAGCTGAGCGAGTGTCTCC<br>R: GATCAGCTCGGGCACTTTAG	229	1.98

[1]F = forward primer; R = reverse primer.
[2]The PCR efficiency was determined by [$10^{(-1/slope)}$]; the slope was obtained by the 7-point standard curve (with a minimum R^2 of 0.99).
[3] Primers for real-time PCR of mouse.
[4] Primers for real-time PCR of cattle.

Supplementary Table 2: List of differentially expressed genes identified in bovine skeletal muscle at two developmental stages

See Supplementary File 1

Supplementary Table 3: 157 terms that were found to be significantly enriched in the category 'molecular function' according to Gene Ontology analysis

See Supplementary File 2

Supplementary Table 4: 153 terms that were significantly enriched in the category 'cellular component' according to Gene Ontology analysis

See Supplementary File 3

Supplementary Table 5: 287 terms that were significantly enriched in the category 'biological processes' according to Gene Ontology analysis

See Supplementary File 4

Supplementary Table 6: 46 genes related to muscle cell proliferation and differentiation that were significantly differentially expressed in two developmental stages of bovine muscle tissue

See Supplementary File 5
